# Supplementary material for: Comprehensive characterization and validation of a fast‐resolving (1000 Hz) plastic scintillator for ultra‐high dose rate electron dosimetry
Source: Med Phys. 2025 Sep 22;52(10):e70006. doi: 10.1002/mp.70006 (PMC12454735; doi:10.1002/mp.70006)
Supplement: Supplementary file 1 — Supporting Information [file MP-52-0-s001.pdf]

Supplementary Material

1. Measurement setup and beam parameters

Figure S1a shows the default setup of the scintillator measurements for CONV and UHDR irradiation. A piece of EBT-XD film was directly placed under the scintillator probe for UHDR measurements (Fig. S1b).

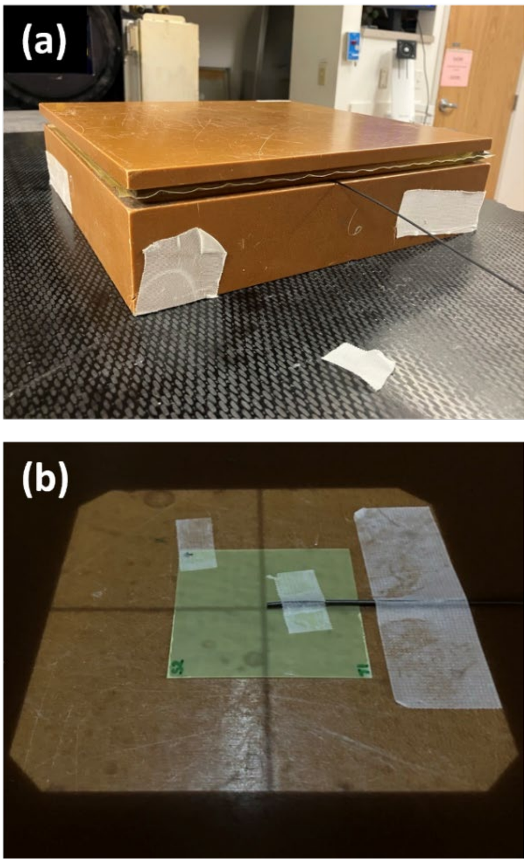

Figure S1. (a) Scintillator measurement setup for CONV and UHDR beams. (b) Relative location of the film and scintillator for UHDR measurements.

Table S1 shows the range of study parameters and the default irradiation setting for CONV and UHDR beams used throughout this study. The CONV irradiation was only used for dose accuracy studies at a sampling frequency of 1 Hz, energy dependence and filed size dependence studies, while the 18 MeV UHDR was used for the remaining tests. Unless otherwise specified, the default values were used throughout this study.

Table S1. Study parameters and default irradiation setting

Symbol	CONV irradiation		UHDR irradiation	
	Study parameters	Default	Study parameters	Default
E	6 and 18 MeV	—	—	18 MeV
SSD	—	100 cm	100–150 cm	100 cm
d	—	2 cm	—	2 cm
FS	2x2–25x25 cm^2	10x10 cm^2	—	10x10 cm^2
PRF	—	—	30–180 Hz	180 Hz
D_{beam}	0–35 Gy	—	0–40 Gy	—
DPP	—	—	0.8–2.3 Gy	2.3 Gy
$\#_{\mathrm{pulse}}$	—	—	2–17	—
t_{pulse}	—	4.5 μs	—	4.5 μs
ADR[a]	—	—	—	414 Gy/s
IDR	—	—	—	5.0 $\times 10^5$ Gy/s

[a]The ADRs of both 6 and 18 MeV CONV beams are 0.1 Gy/s at d_{max}.

Abbreviations: ADR, average dose rate; D_{beam}, absorbed dose; DPP, dose per pulse; d, measurement depth; d_{max}, depth of maximum dose; E, beam energy; FS, field size; IDR, instantaneous dose rate; PRF, pulse repetition frequency; SSD, source to surface distance; t_{pulse}, pulse width; $\#_{\mathrm{pulse}}$, pulse number.

The relationships among MU, pulse number and dose output are shown in Fig. S2. Figure. S2 shows the MU is not linear with respect to pulse number thus output under UHDR conditions as the monitor chamber no longer reliably tracks dose and therefore cannot control dose delivery in a linear manner. The output at the default setup, as measured by the film, remains linear with respect to pulse number.

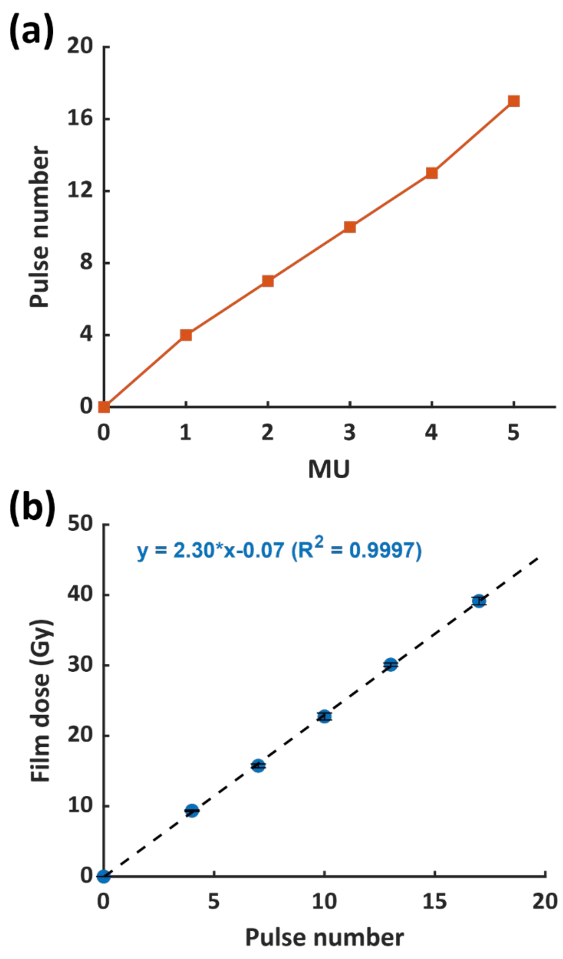

Figure S2. (a) Pulse number vs MU for 18 MeV UHDR. (b) Output measured by film vs pulse number at default setup for 18 MeV UHDR.

2. Verification of field size factors (f_{FS}) measurement against Varian golden beam data

Figure S3 compares the f_{FS} for 6 and 18 MeV electron beams from 6x6 to 25x25 cm^2, measured using the Semiflex 31013 IC at 2 cm depth in solid water, or acquired from golden beam data at d_{ref} in water. The measured f_{FS} and the golden beam data agreed within 1%, validating the accuracy of the IC measurements.

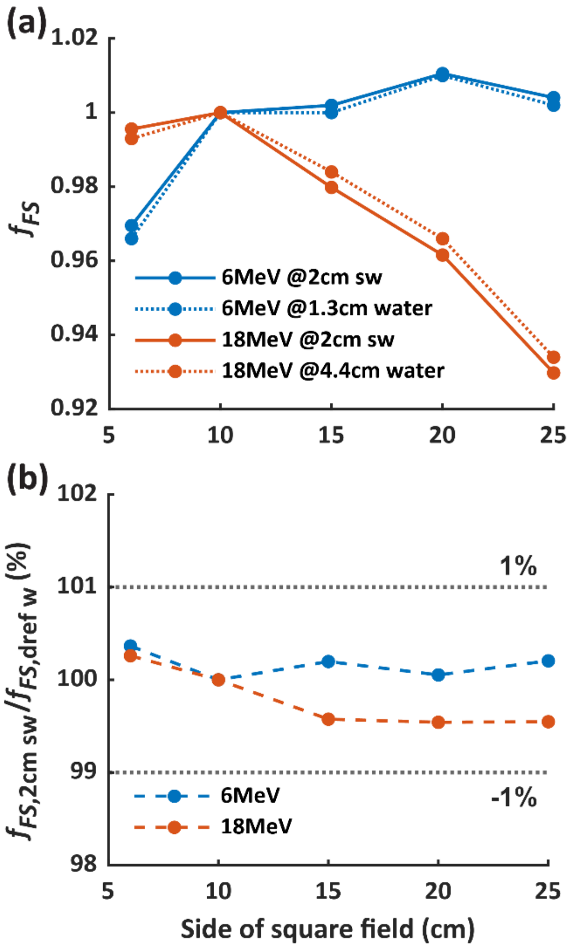

Figure S3. Comparison of filed size factors (f_{FS}) for 6 and 18 MeV CONV. (a) shows the f_{FS} measured with IC at depth of 2 cm, and the f_{FS} acquired from golden beam data at d_{ref} in water. (b) shows the ratio of the f_{FS} measured by the IC to those derived from the golden beam data. Abbreviations: d_{ref}, reference depth; f_{FS}, field size factor; w, water; sw, solid water.

3. Pulse repetition frequency (PRF) measurement with a remote trigger unit

To assess the pulse repetition frequency of the 18 MeV UHDR delivery, the remote trigger unit (RTU, described in Sec 2.5) was positioned outside the radiation field to detect scattered radiation. Four pulses were delivered at 100, 200, 300, and 600 MU/min. As shown in Fig. S4, the PRF corresponded to 30, 60, 90, and 180 Hz, respectively.

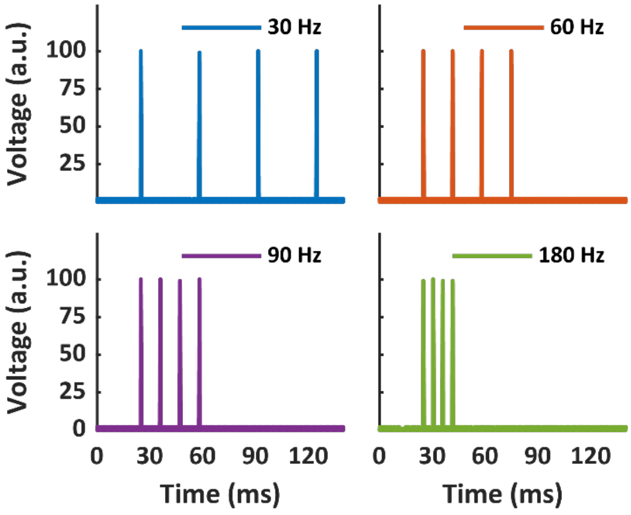

Figure S4. Pulse repetition frequency validated by the remote trigger unit. Abbreviation: a.u., arbitrary unit.

4. Determination of accumulated dose to scintillator using CC13

To determine the accumulated dose delivered to the scintillator, a CC13 ion chamber was placed under the edge of the cone (Fig. S5a) to measure both Bremsstrahlung and scattered radiation. The CC13 readings showed linearity with the dose delivered to the scintillator up to 40 Gy (Fig. S5b).

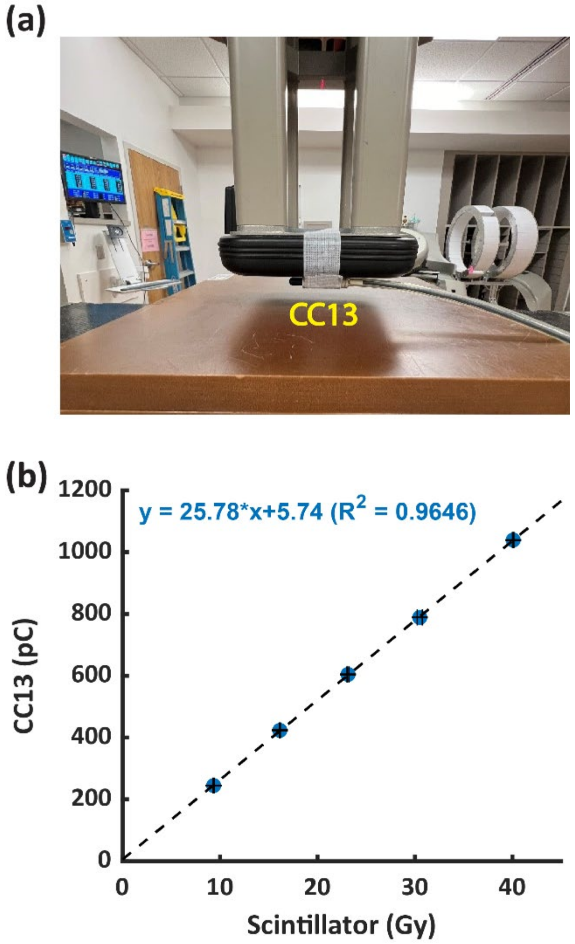

Figure S5. (a) CC13 placed under the edge of the cone. (b) CC13 reading vs scintillator measured dose.

5. Validation of the area under curve (AUC) of PMT signal with respect to dose

Figure S6 shows the AUC of the PMT-fiber optic signal after measuring 4–17 pulses of 18 MeV UHDR, delivered with the default setting (Table S1), in comparison to the film-measured dose. The AUCs of both the full waveform and the segment from signal onset to peak (Fig. 7a) of the total delivered pulses follow a linear relationship with dose, indicating that scattered radiation is proportional to the delivered dose.

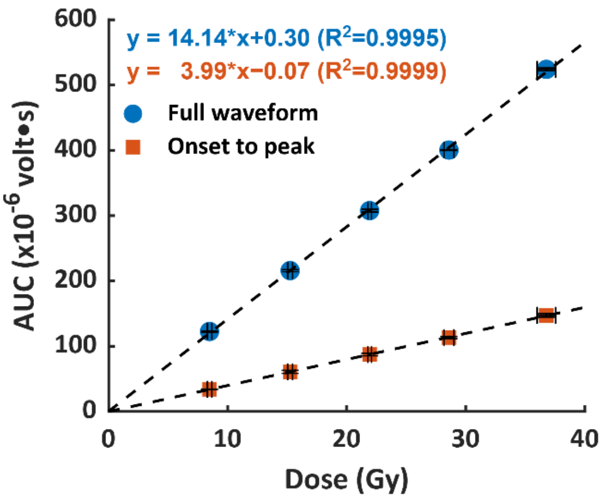

Figure S6. AUC of the PMT-fiber optic signal vs delivered dose. Abbreviation: AUC, area under curve.
